# Transcriptome Analysis Comparison of Lipid Biosynthesis in the Leaves and Developing Seeds of *Brassica napus*

**DOI:** 10.1371/journal.pone.0126250

**Published:** 2015-05-12

**Authors:** Jie Chen, Ren-Ke Tan, Xiao-Juan Guo, Zheng-Li Fu, Zheng Wang, Zhi-Yan Zhang, Xiao-Li Tan

**Affiliations:** Institute of Life Sciences, Jiangsu University, Zhenjiang, P. R. China; Huazhong University of Science and Technology, CHINA

## Abstract

*Brassica napus* seed is a lipid storage organ containing approximately 40% oil, while its leaves contain many kinds of lipids for many biological roles, but the overall amounts are less than in seeds. Thus, lipid biosynthesis in the developing seeds and the leaves is strictly regulated which results the final difference of lipids. However, there are few reports about the molecular mechanism controlling the difference in lipid biosynthesis between developing seeds and leaves. In this study, we tried to uncover this mechanism by analyzing the transcriptome data for lipid biosynthesis. The transcriptome data were *de novo* assembled and a total of 47216 unigenes were obtained, which had an N50 length and median of 1271 and 755 bp, respectively. Among these unigenes, 36368 (about 77.02%) were annotated and there were 109 up-regulated unigenes and 72 down-regulated unigenes in the developing seeds lipid synthetic pathway after comparing with leaves. In the oleic acid pathway, 23 unigenes were up-regulated and four unigenes were down-regulated. During triacylglycerol (TAG) synthesis, the key unigenes were all up-regulated, such as phosphatidate phosphatase and diacylglycerol O-acyltransferase. During palmitic acid, palmitoleic acid, stearic acid, linoleic acid and linolenic acid synthesis in leaves, the unigenes were nearly all up-regulated, which indicated that the biosynthesis of these particular fatty acids were more important in leaves. In the developing seeds, almost all the unigenes in the ABI3VP1, RKD, CPP, E2F-DP, GRF, JUMONJI, MYB-related, PHD and REM transcript factorfamilies were up-regulated, which helped us to discern the regulation mechanism underlying lipid biosynthesis. The differential up/down-regulation of the genes and TFs involved in lipid biosynthesis in developing seeds and leaves provided direct evidence that allowed us to map the network that regulates lipid biosynthesis, and the identification of new TFs that are up-regulated in developing seeds will help us to further elucidate the lipids biosynthesis pathway in developing seeds and leaves.

## Introduction

Lipids have many important biological functions, including storing energy, signaling and acting as structural components of cell membranes [1, 2]. Lipids, which are important biological macromolecules, occur in different forms, including fats, waxes, sterols, fat-soluble vitamins (such as vitamins A, D, E and K), monoglycerides, diglycerides, triglycerides, phospholipids and glycolipids. In plants, the lipid type and contents differ between the leaf and seed: plant seed is the oil-rich organ, whereas leaf mainly contains glycolipids and phospholipids.

The leaf is where photosynthesis occurs, which provides plants with the large amounts of carbohydrates and energy needed for growth and development. Glycolipids and phospholipids are the main components of the photosynthetic membranes in plant chloroplast envelopes and stroma [3]. They are also involved in the formation of the photosynthetic membranes and are part of the photosynthetic complexes [4–7]. The main lipid in leaves is monogalactosyldiacylglycerol (MGDG), followed by digalactosyldiacylglycerol (DGDG), phosphatidylglycerol (PG) and phosphatidylcholine (PC). There is also a small amount of triacylglycerol (TAG) [8]. However, in the seed, as the storage organ, most of the lipid is triacylglycerol, which is stored in the oil body (OB). Neutral lipids account for a large percentage of the oil in the seeds of rape, mustard, cotton, flax, maize, peanut and sesame [9]. Furthermore, *Arabidopsis* studies have revealed that there is a difference in lipid content and type between leaves and seeds, with 52.46% glycerolipids, 24.60% chlorophyll, 4.92% cutin monomers, 3.28% sphingolipids, 3.28% wax and 11.48% others present in leaves, and 94% storage lipids, 5% membrane lipids and 1% surface lipids present in seeds [3]. The total lipid content in seeds was more than in leaves and accounted for 37% of the total dry weight of seeds, but only 6.1% of the total dry weight in leaves. There was a difference in storage lipid content between the leaves and seeds and a significant difference in fatty acid contents. In *Arabidopsis*, the highest fatty acid content in seeds was 18:2, followed by 20:1, 18:3, 18:1, 16:0 [10], whereas the highest fatty acid content found in leaves was 18:3, followed by 18:2, 16:0, 16:3 [11]. In *Brassica napus*, the highest fatty acid content in seeds was 18:1, followed by 18:2, 18:3, 16:0 and others [12]. This may be due to the different functions of fatty acids in leaves and seeds. The fatty acids in leaves may be involved in the formation of membrane lipid structure, whereas the fatty acids in seeds may act as storage lipids [3].

*Brassica napus* is one of the most important edible oilseed crops in the world and produces considerable amounts of edible oil for human consumption. Research by United States Department of Agriculture (USDA) showed that the rapeseed provided more and more oil for human consumption, ranging from 24916 thousand metric tons to 26946 thousand metric tons in three years (http://www.usda.gov/wps/portal/usda/usdahome). Rapeseed breeding [13, 14] has produced varieties with zero seed erucic acid and low seed glucosinolate levels. It has also produced rapeseed with high oleic acid contents (78% to 88%), which has nutritional and health benefits, and high oil contents (40% to 45%) [15]. Many genes related to oleic acid and oil biosynthesis and regulation have been elucidated. In the prokaryotic fatty acid biosynthesis pathway, fatty acid desaturase 2 (FAD2) is involved in the regulation of oleic acid, linoleic acid and linolenic acid biosynthesis [16, 17], and acetyl-CoA carboxylase (ACCase) and the fatty acid synthase complex (FAS) are the key enzymes in the fatty acid synthesis [18, 19]. In TAG biosynthesis pathway, glycerol-3-phosphate acyltransferase 4 (GPAT4) and acyl CoA binding protein (ACBP) were involved in the regulation of oil content and fatty acid composition [12, 20]. 1-acyl-sn-glycerol-3-phosphate acyltransferase (LPAT) [21, 22], diacylglycerol O-acyltransferase (DGAT) and phosphatidate phosphatase (PP) [23, 24] were involved in TAG synthesis [25, 26]. Some transcription factors (TFs) also play key roles in lipid biosynthesis. Previous studies have revealed that ABI3, LEC1, LEC2, FUS3, WRI1, AP2, GL2, EMF2, HSI2, L1L, PKL, FIE and SWN acted as the regulator in fatty acid and TAG synthesis [27, 28].

Next generation sequencing (NGS) enables us to obtain genetic information [29–31]. Many plants have been sequenced and annotated using NGS, such as *B*. *rapa* [32] and *B*. *oleracea* [33], which are the species from which the *Brassica napus* originated, and other oil producing plants, such as palm [34], peanut [35], sesame [30], safflower [36], rape [37, 38], jatropha [39] and yellow horn [40]. Lipid contents differ significantly between the leaves and seeds in *Brassica napus* and lipid biosynthesis and regulation are also different. Although the *de novo* biosynthesis of fatty acids and lipids is now well understood, much less is known about how plants produce the different amounts and types of fatty acid and lipids between seeds and leaves in *B*. *napus* through the regulation of gene expressions. In this study, we compared the developing seeds and leaves transcriptome in *B*. *napus*, which revealed how seeds were able to store so much TAG and offered us clues on how to improve the content of specific lipids in seeds.

## Materials and Methods

### Plant material

*B*. *napus* cv Ninyou 12 was used as the material. The leaves at the stage when plant had 4–5 leaves, at the top two position were collected as the sample and developing seeds at 25 days after pollination (DAP) were harvested in the field and immediately frozen in liquid nitrogen and stored at—70°C for RNA extraction. Hereafter, seeds refer to 25DAP seeds unless special illustration. The experimental material was planted in Jiangsu University, and was specially used as the experimental research. And *Brassica napus*, has been used as the research object with no dangerous and harmful to the land and crop. The measurement of the fatty acid and TAG contents in leaves followed a previously reported method [41, 42], and mature seeds oil contents were measured using near infrared-reflectance spectroscopy (NIRS) [43, 44].

### RNA extraction, library construction and RNA-seq

Total RNA of the collected leaves and 25 DAP seeds were extracted using TRIzol Reagent (Life technologies, Shang hai, USA) according to the manufacturer’s instructions. The extracted RNA was qualified and quantified using a OneDrop OD-1000+ spectrophotometer (RockGene, Shanghai, China) and the samples showed a 260/280 nm ratio between 1.8 and 2.2, and an OD260/230 > 1.0, which were within the requirements of Beijing Biomarker Technologies (http://www.biomarker.com.cn/index.php).

The mRNA-seq library was constructed using Illumina’s TruSeq RNA Sample Preparation Kit (Illumina lnc, San Diego, CA, USA), and the isolation of mRNA, fragment interruption and RNA-Seq were performed by the company according to their standard protocol. Finally, the mRNA-seq library was constructed for sequencing using the Illumina HiSeqTM 2000 sequencing platform.

### Analysis of transcriptome sequencing results

The raw reads were first filtered by discarding the reads with adaptor contamination, low-quality sequences (reads with ambiguous ‘N’ bases), and reads with more than 10% Q < 20 bases. Then the clean reads were assembled into contigs using the Trinity program [45], which efficiently reconstructed full-length transcripts across a broad range of expression levels and sequencing depths. Subsequently, the contigs were linked into transcripts according to the paired-end information of the sequences, and the transcripts were clustered based on nucleotide sequence identity. The longest transcripts in the cluster units were regarded as unigenes in order to eliminate redundant sequences, and then the unigenes were combined to produce the final assembly used for annotation. The unigenes information was deposited in the Sequence Read Archive (SRA) database in NCBI (Accession number, SRR1916242).

To understand their functions, the unigenes were annotated using BLASTx alignment, with an E-value cut-off of 10^–5^, against the NCBI non-redundant (NR) database, and the UniProt/Swiss-Prot, Kyoto Encyclopedia of Genes and Genomes (KEGG), Cluster of Orthologous Groups of proteins (COG) and Gene Ontology (GO) databases.

The RPKM (Reads Per Kilobase per Million mapped reads) method was used to calculate unigenes’ expression [46]. The RPKM method is able to reflect the molar concentration of a transcript by normalizing for RNA length and for the total read number. We compared the unigenes expressions using their RPKM values.

### Detection of TFs in the transcriptome data

To detect TFs, we performed a BLAST search for all unigenes against th*e AGRIS (Arabidopsis* Gene Regulatory Information Server) database with an e-value cut off of 10^–5^ [47].

### Quantitative real-time PCR analysis

The selected differentially expressed transcript factors were confirmed through qRT-PCR using ABI 7300 Real-Time PCR Detection System (Applied Biosystems, Foster City, CA, USA) with SYBR Premix Ex TaqTM II (TaKaRa, Tokyo, Japan). First, we used RNase-free DNase I to remove residual trace amounts of DNA before cDNA synthesis, according to the manufacturer’s instruction (Thermo Scientific, Waltham, MA, USA). The synthesis of first strand of cDNA was according to the manufacturer’s instructions of the RevertAid First Strand cDNA Synthesis Kit (Thermo Scientific) from 2μg of total RNA in a 20μL reaction using oligodT primers. Then the cDNA sets were diluted 1:10 with nuclease-free water and used for qPCR analysis. Each 20μL reaction mixture contained 10μL of 2×SYBR Premix Ex TaqTM, 2μL of diluted cDNA, 2μL of each primer (2μM), 0.4μL of ROX Reference Dye (50×) and 3.6μL of double distilled water. The qPCR cycling conditions were as follows: 95°C for 30s; followed by 40 cycles of 95°C for 10s, the respective annealing temperature for 30s and 72°C for 27s in PCR strip tubes (Axygen, Union City, CA, USA). We employed probes specific for the TIP41 [48] as references to analyze the expression level of TFs between leaves and seeds, and each reaction was performed three repeats.

## Results and Discussion

### Comparison of fatty acid contents between seeds and leaves

Seeds and leaves have considerable morphological differences. The fatty acid contents in seeds and leaves are also different (Table 1). The seeds contained oleic acid (59.14%), linoleic acid (23.43%), linolenic acid (9.18%), saturated fatty acids (6.61%) and others (1.64%). However, in the leaves, the largest fatty acid component was linolenic acid (47.55%), followed by saturated fatty acids (20.15%), linoleic acid (10.62%), oleic acid (4.85%) and others (16.82%). Hence, these data showed the big difference between the components in leaves and seeds. We tried to discern the mechanism controlling lipid biosynthesis between the seeds and leaves by their transcriptome.

**Table 1:** The content of different fatty acids between leaves and 25DAP seeds.

Component	oleic acid	linoleic acid	linolenic acid	saturated fatty acids	Others
Leaves(mol%)	4.85	10.62	47.55	20.15	16.82
Seeds(mol%)	59.14	23.43	9.18	6.61	1.64

### The raw data of leaves and seeds transcriptome sequencing

**Fig 1:**
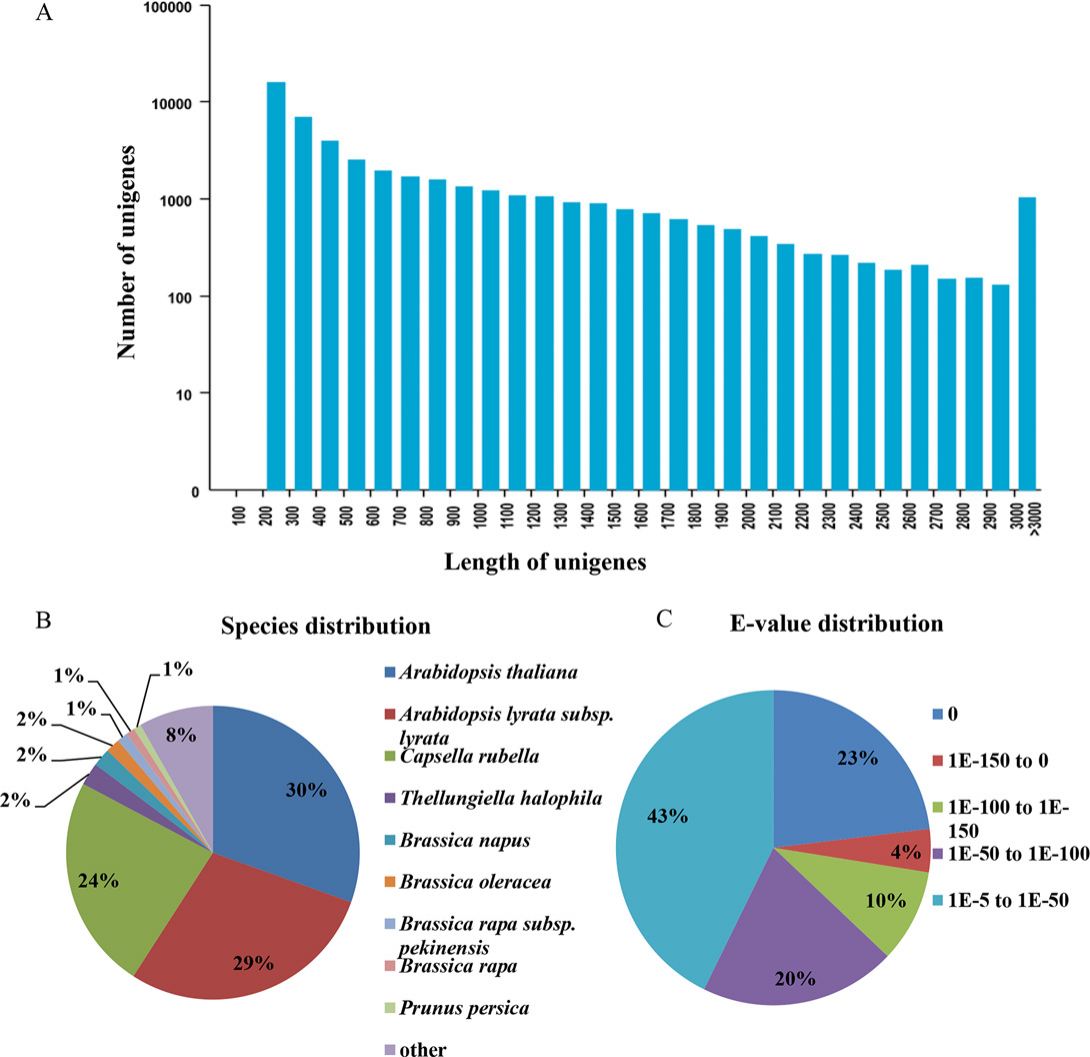
Unigenes functional annotation results. (A) Distribution of unigenes in length. (B) Top species distribution for BLASTx matches for *B*. *napus* unigenes using the following order of priority: NR and Swiss-Prot. (C) E-value distribution of top BLASTx hits for each unigene.

By sequencing, the leaves sample produced 27086293 reads and the seeds sample produced 40496936 reads (Table 2). The average quality value was ≥ 20 for 100% of the cycle with a near zero ambiguous “N”. The Q30 percentage exceeded 80% and the GC content 48.67% and 48.68% for the leaves and seeds, respectively, which suggested that the sequencing was highly accurate and reliable. After the removal of adaptor sequences and the exclusion of contaminated or short reads, the high-quality reads were assembled into 10949964 contigs with a mean length of 39.2 bp, 114337 transcripts with a mean length of 1008.59 bp and 47216 unigenes with a mean length of 755.65 bp (Table 3) using the Trinity *de novo* assembly program [45]. Out of these 47216 unigenes, 11528 unigenes were ≥ 1000 bp and accounted for 24.41% of the total. The size distribution of all the unigenes is shown in Fig 1A. These results showed that the throughput and sequencing quality were high enough for the following analyses.

**Table 2:** The raw data of transcriptome between leaves and 25DAP seeds in *Brassica napus*.

SampleID	Total reads	Total nucleotides(bp)	GC(%)	N(%)	Q20%	CycleQ20%	Q30%
Leaves	27086293	5457985744	48.67	0.08	89.83	100	80.74
Seeds	40496936	8098613808	48.68	0	89.3	100	80.58

**Table 3:** The de novo assembly of raw data.

Length range	Contigs	Transcripts	Unigenes
200–300	10910319(99.64%)	21416(18.73%)	15816(33.50%)
300–500	16636(0.15%)	18974(16.59%)	10863(23.01%)
500–1000	11316(0.10%)	28213(24.68%)	9009(19.08%)
1000–2000	8465(0.08%)	33325(29.15%)	8192(17.35%)
2000+	3228(0.03%)	12409(10.85%)	3336(7.07%)
Total number	10949964	114337	47216
Total length	429283622	115318903	35678684
N50 length	37	1459	1272
Mean length	39.2	1008.59	755.65

### Functional annotation

According to the results of functional annotation (Table 4), there were 36176 (76.62%) unigenes homologous proteins in the Nr protein database and 26964 (57.11%) unigenes similar to proteins in the Swiss-Prot database. And, 29780 (63.07%), 9394 (19.90%) and 8123 (17.20%) unigenes had significant matches with sequences in the GO, COG and KEGG databases, respectively. In total, 36368 (77.02%) unigenes were successfully annotated using the Nr, Swiss-Prot, GO, COG and KEGG databases. However, the remaining unmapped unigenes (22.98%) were not annotated in these databases, which could be attributable to the short sequence reads generated by the sequencing technology or the relatively short sequences of the resulting unigenes lacked conserved functional domains [49].

**Table 4:** The functional annotation of unigenes in COG, GO, KEGG, Swissprot and Nr database.

Anno Database	Annotated Number	300< = length<1000	length> = 1000
COG_Annotation	9394(19.90%)	3130	4983
GO_Annotation	29780(63.07%)	12642	9629
KEGG_Annotation	8123(17.20%)	3215	2828
Swissprot_Annotation	26964(57.11%)	10892	9902
nr_Annotation	36176(76.62%)	15660	11154
All_Annotated	36368(77.02%)	15722	11168

To identify the species specificity of the unigenes (36176) annotated in the Nr database, we matched these unigenes and found that all the unigenes were found in at least one species, with unigenes from *Arabidopsis thaliana* (11023, 30.47%), *Arabidopsis lyrata subsp*. *Lyrata* (10362, 28.64%), *Capsella rubella* (8553, 23.64%), *Thellungiella halophile* (905, 2.50%), *Brassica napus* (720, 1.99%), *Brassica oleracea* (567, 1.56%), *Brassica rapa subsp*. *Pekinensis* (431, 1.19%), *Brassica rapa* (345, 0.95%), *Prunus persica* (300, 0.83%) and other species (2970, 8.21%) (Fig 1B). The little proportion of unigenes belonging to *B*. *napus* might be the results of searching in public databases which contain few data of *B*. *napus*. We also found that 57.26% unigenes had an E-value of less than 1.0E^-50^, and there was a very strong homology among these aligned unigenes. The remaining 42.74% unigenes had an E-value of between 1.0E^-5^ to 1.0E^-50^ (Fig 1C).

### GO, COG and KEGG classification analysis

**Fig 2:**
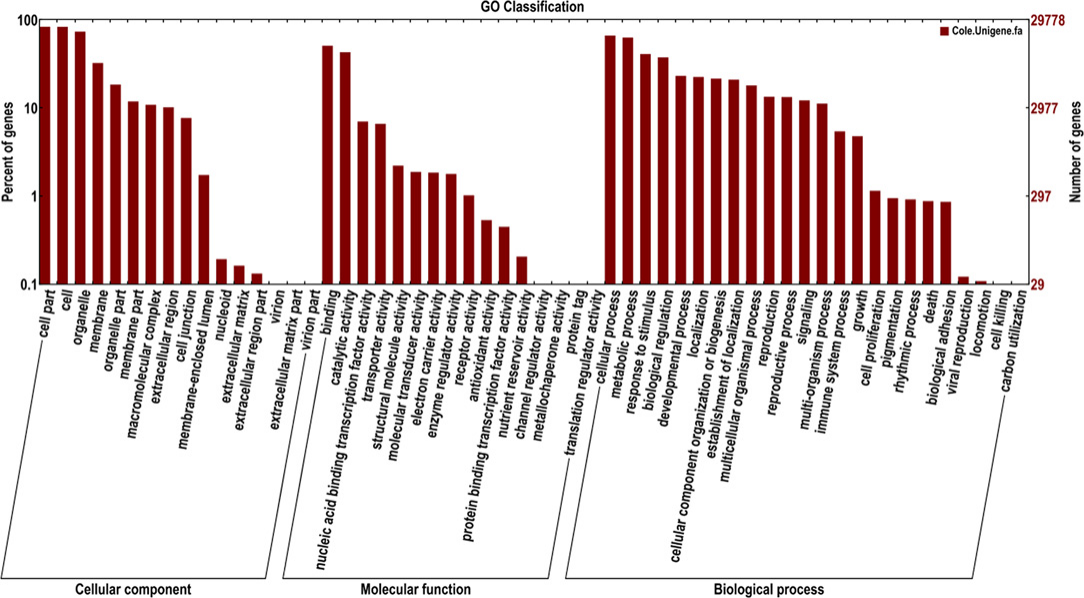
Gene Ontology (GO) categories assigined to the *B*. *napus* unigenes.

To further predict and classify the function of annotated unigenes, we used the sequences of these unigenes to search for genes with GO assignments, COG classifications and KEGG pathway assignments. First, we performed a Gene Ontology (GO) [50] analysis based on their Nr annotation, which revealed the cellular component, molecular function and biological process unigenes, based on sequence homology. Among the unigenes, 29780 were assigned into three main GO functional categories and then were divided into 56 sub-categories, among which many unigenes were assigned to one or more sub-categories (Fig 2). The largest category was biological process containing 112676 unigenes, followed by cellular component (99279) and molecular function (34902). The biological process category contained 24 sub-categories and two of the biggest sub-categories were “cellular process” and “metabolic process”, which contained 19705 and 18703 unigenes, respectively, which suggested that these unigenes were enriched in the *B*. *napus* transcriptome libraries. The second category, cellular component, was divided into 16 sub-categories and three of the largest sub-categories were “cell part”, “cell” and “organelle”, which contained 24824, 24784 and 21854 unigenes, respectively. The last category, molecular function, was categorized into 16 GO sub-categories and the two largest sub-categories were “binding” and “catalytic activity”, with 15115 and 12754 unigenes, respectively.

**Fig 3:**
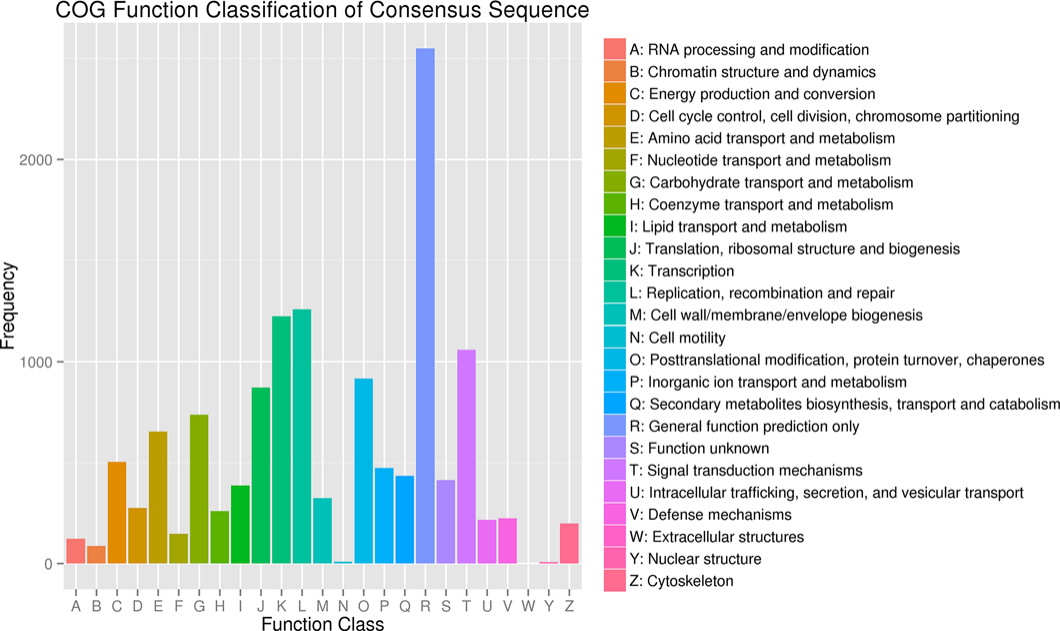
Clusters of orthologous groups (COG) classifications of *B*. *napus* unigenes.

Then, we performed a COG analysis of all the unigenes for functional prediction and classification. A total of 9394 unigenes sequences showed a hit with the Nr database and could be assigned to COG classifications that were functionally clustered into 24 COG categories with no unigenes involved in the “Extracellular structures” category. Among these categories, the cluster for “General function prediction only” was the largest group containing 2550 unigenes (27.14%), followed by “Replication, recombination and repair” (1258, 13.39%), “Transcription” (1223, 13.02%), “Signal transduction mechanisms” (1059, 11.27%), “Post-translational modification, protein turnover chaperones” (917, 9.76%), “Translation, ribosomal structure and biogenesis” (872, 9.28%) and “Carbohydrate transport and metabolism” (737, 7.85%). Only a few unigenes were assigned to the two smallest categories, “Cell motility” and “Nuclear structure” (9 and 7 unigenes, respectively) (Fig 3).

Finally, in order to better understand the biological pathways in *B*. *napus*, we used the KEGG [51] database to categorize gene functions with an emphasis on biological pathways. The results showed that a total of 8123 unigenes were assigned to 121 pathways (Table 4; S1 Table). The number of unigenes involved in these 121 pathways was 8515 instead of 8123, which suggested that some unigenes might be involved in more than one KEGG pathway, such as unigene “c18488.graph_c0” which is involved in the glycerolipid metabolism, galactose metabolism, sphingolipid metabolism and glycosphingolipid biosynthesis-globo series pathways. Among the 121 pathways, the largest pathway was Plant hormone signal transduction, which contained 384 unigenes, followed by Ribosome (334), Plant-pathogen interaction (252), Protein processing in endoplasmic reticulum (239) and RNA transport (234), etc. The smallest pathway was Anthocyanin biosynthesis, which only contained one unigene (S1 Table). When we concentrated on fatty acid and lipid biosynthesis and metabolism, we found that there were 95 unigenes for glycerophospholipid metabolism, 73 for fatty acid metabolism, 64 for glycerolipid metabolism, 56 for biosynthesis of unsaturated fatty acids, 36 for fatty acid biosynthesis, 35 for pantothenate and CoA biosynthesis, 27 for linoleic acid metabolism, 23 for arachidonic acid metabolism and 8 for fatty acid elongation in mitochondria. These results will provide precise and more targeted information for further analysis.

### Differentially expressed genes (DEG) analysis

**Fig 4:**
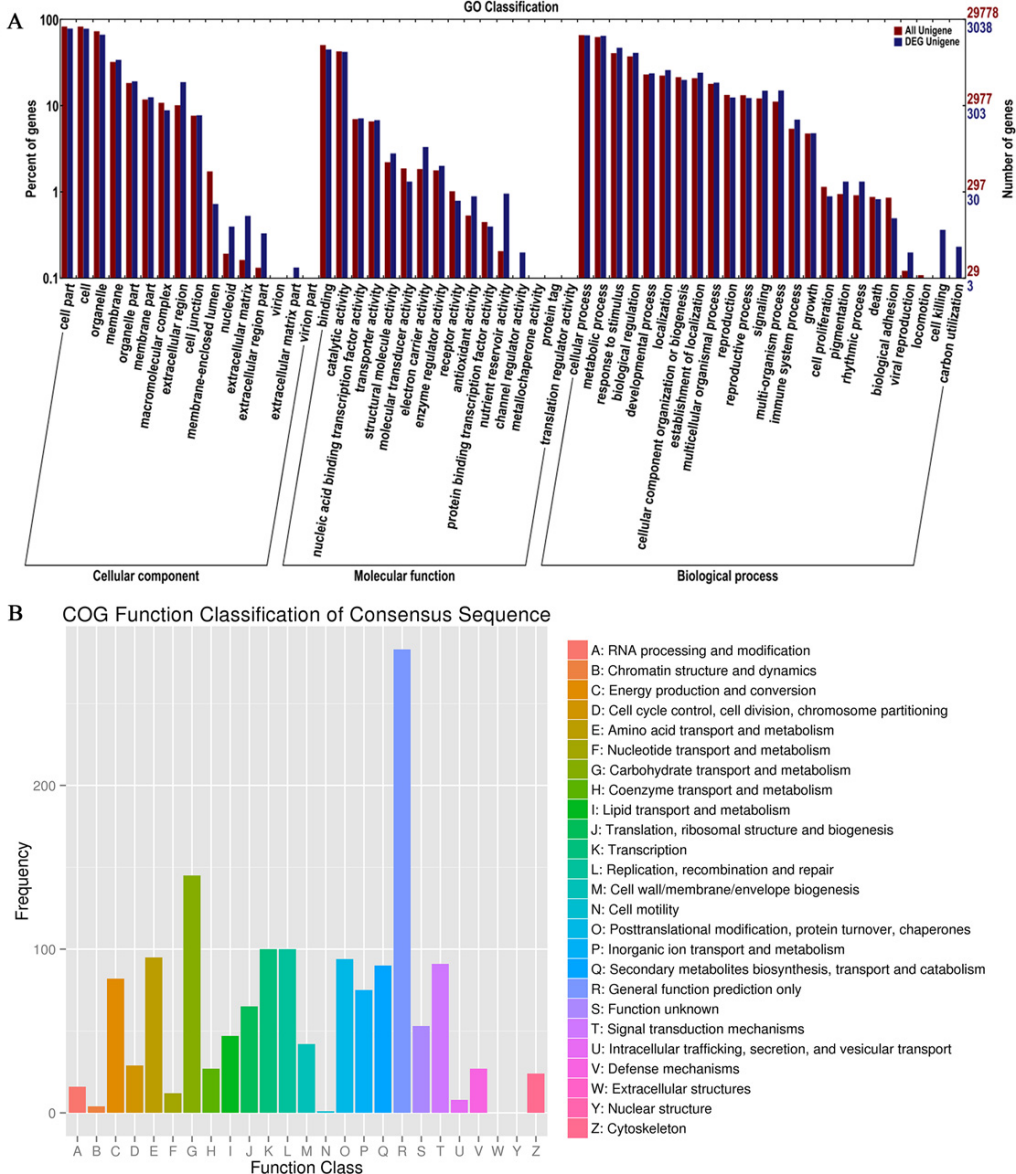
Function classification of Differential Expression Genes (DEG) in *B*. *napus*. (A) Gene ontology (GO) classification of DEG. (B) Clusters of orthologous groups (COG) classifications of DEG.

The difference in lipid content between seeds and leaves might be caused by different genes expression. So we performed a differentially expressed genes (DEG) analysis and found that there were 4544 unigenes that were differentially expressed. We then performed GO and COG classification analyses to identify the function of these differentially expressed genes (Fig 4). In the GO classification analysis, 4544 unigenes were assigned to three main GO functional categories and then were divided into 56 sub-categories, among which many unigenes were assigned to more than one sub-category. Then we calculated the percentage of DEG involved in each sub-category (S2 Table). The largest percentage of sub-category in the “cellular component” category was extracellular matrix part (DEG accounting for 80.00% of all unigenes involved in this category), followed by extracellular matrix (33.33%), extracellular region part (25.64%) and nucleoid (21.05%). The largest percentage of sub-category in “molecular function” was channel regulator activity (50%), followed by nutrient reservoir activity (47.54%) and protein tag (22.22%). The largest percentage of sub-category in “biological process” was cell killing (57.89%), followed by utilization (46.67%). These results indicated that the difference in lipid content between the seeds and leaves might be due to the differential expression of genes in these nine sub-categories, which would provide a direction for further analysis.

The COG function classification analysis showed that the DEG were distributed across 24 COG categories. We also calculated the percentage of DEG in each category (S3 Table) and found that the largest percentage of category was “Secondary metabolites biosynthesis, transport and catabolism” (20.69%), followed by “Carbohydrate transport and metabolism” (19.67%), “Energy production and conversion” (16.27%) and “Inorganic ion transport and metabolism” (15.82%). Many unigenes were differentially expressed in these categories, which might result in the different lipid contents seen in the seeds and leaves.

### Genes related to fatty acid biosynthesis in *B*. *napus*

Fatty acids are stored as a form of TAG and their biosynthesis pathway can be divided into three steps in nearly all oil plants [52]. The first step is *de novo* fatty acid synthesis. In plants, *de novo* fatty acid synthesis occurs in the plastid instead of the cytosol and is catalyzed mainly by the fatty acid synthase complex (FAS). Furthermore, biosynthesis is not restricted to specific tissues or organs, but occurs in every plant cell [3]. The second step is the synthesis of triacylglycerol (TAG) using the fatty acid and glycerol as substrates. This occurs in the endoplasmic reticulum (ER). Finally, TAG is combined with oil proteins, such as oleosin, caleosin and steroleosin, to form OBs (oil bodies), which are released from the ER into the cytoplasm [53, 54].

**Fig 5:**
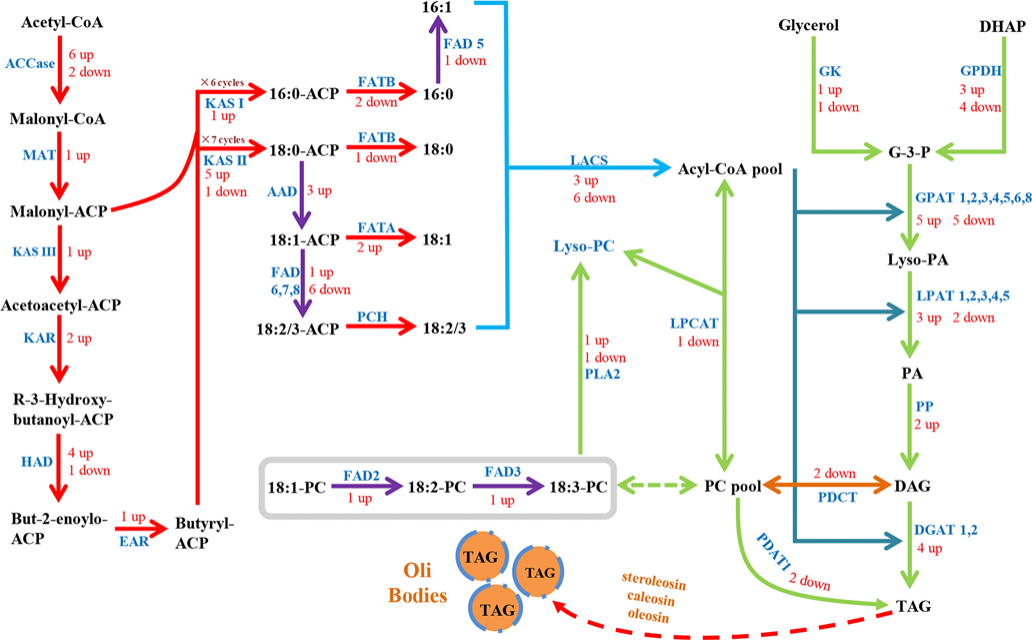
Overview of de novo fatty acid (FA) and triacylglycerol (TAG) biosynthesis pathways. Indentified enzymes include: ACCase, aacetyl-CoA carboxylase carboxyl transferase (EC:6.4.1.2); MAT, Malonyl-CoA-ACP transacylase (EC:2.3.1.39); KAS, 3- oxoacyl ACP synthase (KASI, EC: 2.3.1.41; KASII, EC: 2.3.1.179; KAS III, EC: 2.3.1.180); KAR, 3-oxoacyl ACP reductase (EC:1.1.1.100); HAD, 3R-hydroxymyristoyl ACP dehydrase (EC:4.2.1.-); EAR, enoyl-ACP reductase I (EC:1.3.1.9); FATA/B, fatty acyl-ACP thioesterase A/B (EC:3.1.2.14 3.1.2.-); AAD, acyl-ACP desaturase (EC:1.14.19.2); PCH, palmitoyl-CoA hydrolase (EC:3.1.2.2); LACS, long-chain acyl-CoA synthetase (EC:6.2.1.3); FAD2/6, D12(ω6)-Desaturase (EC:1.14.19.-); FAD3/7/8, D15(ω3)-Desaturase (EC:1.14.19.-); GK, glycerol kinase (EC:2.7.1.30); ATS1/GPAT, glycerol-3-phosphate acyltransferase (EC:2.3.1.15); LPAT, lysophosphatidyl acyltransferase (EC:2.3.1.51); PP, phosphatidate phosphatase (EC:3.1.3.4); DGAT1, diacylglycerol O-acyltransferase 1 (EC:2.3.1.20); PDAT1, phospholipid: diacylglycerol acyltransferase 1 (EC:2.3.1.158); LPCAT, lysophosphatidylcholine acyltransferase (EC:2.3.1.23 2.3.1.67); PLA2, Phospholipase A2 (EC:3.1.1.4). Numbers indicated the numbers of each enzyme, and “up” meant up-regulation comparing 25DAP seeds with leaves, and “down” meant down-regulation. Lipid substrates are abbreviated: 16:0, palmitic acid; 18:0, stearic acid; 18:1, oleic acid; 18:2, linoleic acid.

A manually repeated search based on the KEGG pathway assignment and functional annotation of the unigenes found that 36 unigenes were annotated as encoding ten key enzymes involved in fatty acid biosynthesis and ten unigenes encoding acyl carrier protein (ACP) (Table 5; S4 Table). Based on these identified enzymes, we reconstructed the fatty acid biosynthesis pathway by referencing previous reports (Fig 5) [3, 40]. The first committed step in fatty acid synthesis is the formation of malonyl-CoA from acetyl-CoA, which is catalyzed by acetyl-CoA carboxylase (ACCase, EC: 6.4.1.2) [18]. We identified ten unigenes that were involved in encoding four subunits of this enzyme (four for biotin carboxyl carrier protein, three for biotin carboxylase, one for α-carboxyltransferase and two for β-carboxyltransferase). Among these ten unigenes, six were up-regulated in seeds compared to leaves, two were down-regulated and two were unchanged, which suggested that this critical process would provide more substrates for the fatty acid synthesis in seeds than in leaves. Next, the fatty acids are grown by a series of condensation reactions with malony-CoA. The ACP-bound acyl chain grows by consecutively adding two carbon units per cycle over six or seven cycles to form 16:0-ACP or 18:0-ACP. This process is catalyzed by the fatty acid synthase complex (FAS), which is composed of five components encoded by 20 unigenes. Two unigenes encoded malonyl-CoA-ACP transacylase (MAT, EC: 2.3.1.39), ten encoded 3-oxoacyl-ACP-synthase (KAS; one encoded KAS I (EC: 2.3.1.41), six encoded KAS II (EC: 2.3.1.179), three encoded KAS III (EC: 2.3.1.180)), two encoded 3-oxoacyl-ACP reductase (KAR, EC: 1.1.1.100), five encoded hydroxyacyl-acyl-ACP dehydratase (HAD, EC: 4.2.1.-) and one encoded enoyl-ACP reductase (EAR, EC: 1.3.1.9). Besides the five components of FAS, the acyl carrier protein (ACP), as a cofactor, is also an essential part of FAS. We found that ten unigenes encoded ACP. From Fig 5, we can see that almost all the unigenes encoding FAS were up-regulated in the *de novo* synthesis of 16:0-ACP and 18:0-ACP in seeds compared to the leaves. Only two unigenes were down-regulated, which meant that the up-regulation of these unigenes would increase the fatty acid content in seeds. After this, the 16:0-ACP and 18:0-ACP are catalyzed by acyl-ACP desaturase (AAD, EC: 1.14.19.2) to form 16:1-ACP and 18:1-ACP [55], or 18:0-ACP is desaturated by fatty acid desaturase (FAD, EC: 1.14.19.-) to form 18:2/3-ACP. Finally, under the control of acyl-ACP thioesterase (FAT, EC: 3.1.2.14 3.1.2.-) and palmitoyl-CoA hydrolase (PCH, EC: 3.1.2.2), free fatty acids are released from the acyl carrier protein (ACP). Three unigenes that encoded AAD were all up-regulated, which suggested that *B*. *napus* tends to produce unsaturated fatty acid in the seeds. Four unigenes that encoded FATB were identified, of which three unigenes were down-regulated to form 16:0 palmitic acid and 18:0 stearic acid. Two unigenes that encoded FATA were all up-regulated to form 18:1oleic acid, which was the most common fatty acid in *B*. *napus* seeds (59.14%).

**Table 5:** Enzymes/protein related to FA biosynthesis and metabolism identified by annotation of the *B*. *napus* unigenes.

Symbol	Enzymes/Protein	EC Number	Number of unigenes	Up/Down(numbers of unigenes)
**Fatty acid biosynthesis**				
ACP	Acyl carrier protein		10	3up5down
accB/bccp	acetyl-CoA carboxylase biotin carboxyl carrier protein		4	3up1down
ACC	Acetyl-CoA carboxylase 1/Biotin carboxylase	EC:6.4.1.2	3	2up
accA/accD	Acetyl-coenzyme A carboxylase carboxyl transferase subunit alpha/beta	EC:6.4.1.2	3	1up1down
MAT	Malonyl-CoA-acyl carrier protein transacylase	EC:2.3.1.39	2	1up
KAS III	3-oxoacyl (ketoacyl)-[acyl-carrier-protein] synthase III	EC:2.3.1.180	3	1up
KAS II	3-oxoacyl-[acyl-carrier-protein] synthase II	EC:2.3.1.179	6	5up1down
KAS I	3-oxoacyl-[acyl-carrier-protein] synthase I	EC:2.3.1.41	1	1up
KAR	3-oxoacyl-[acyl-carrier-protein] reductase	EC:1.1.1.100	2	2up
HAD	hydroxyacyl-acyl-ACP dehydratase	EC:4.2.1.-	5	4up1down
EAR	Enoyl-[acyl-carrier-protein] reductase [NADH]	EC:1.3.1.9	1	1up
FATA	Oleoyl-acyl carrier protein thioesterase	EC:3.1.2.14 3.1.2.-	2	2up
FATB	Palmitoyl/stearoyl-acyl carrier protein thioesterase	EC:3.1.2.14 3.1.2.-	4	3down
**Fatty acid elongation**				
KCS1	3-ketoacyl-CoA synthase	EC:2.3.1.-	21	3up9down
KCR2(KR)	Ketoacyl-CoA Reductase	EC:1.1.1.-	3	1down
HACD(PSH1)	Hydroxyacyl-CoA Dehydratase	EC:4.2.1.-	7	3up
ECR	enoyl-CoA reductase	EC:1.3.1.38	2	2up
PPT	Palmitoyl-protein thioesterase 1	EC:3.1.2.22	6	3up1down
PCH	palmitoyl-CoA hydrolase	EC:3.1.2.2	1	
**Fatty acid desaturation**				
AAD	Acyl-ACP desaturase	EC:1.14.19.2	3	3up
FAD2	Omega-6 fatty acid desaturase, endoplasmic reticulum	EC:1.14.19.-	1	1up
FAD3	Omega-3 fatty acid desaturase, endoplasmic reticulum	EC:1.14.19.-	1	1up
FAD5	Palmitoyl-monogalactosyldiacylglycerol delta(δ)-7 desaturase, chloroplastic	EC:1.14.19.-	4	1down
FAD6	Omega-6 fatty acid desaturase, chloroplastic	EC:1.14.19.-	2	2down
FAD7	Omega-3 fatty acid desaturase, chloroplastic	EC:1.14.19.-	6	1up2down
FAD8	Omega-3 fatty acid desaturase, chloroplastic	EC:1.14.19.-	2	2down
**Fatty acid metabolism**				
ACAT	acetyl-CoA acyltransferase	EC:2.3.1.16	1	1up
AACT	Acetyl-CoA C-acetyltransferase	EC:2.3.1.9	4	2up
ACAD	Acyl-CoA dehydrogenase	EC:1.3.99.3	3	1up1down
ACOX	acyl-coenzyme A oxidase	EC:1.3.3.6	11	1up5down
LACS	long-chain acyl-CoA synthetase	EC:6.2.1.3	10	3up6down
ADH	Alcohol dehydrogenase	EC:1.1.1.1	18	5up5down
ALDH	Aldehyde dehydrogenase	EC:1.2.1.3	23	10up6down
MFP2	enoyl-CoA hydratase/3-hydroxybutyryl-CoA dehydrogenase 2	EC:4.2.1.17 1.1.1.35 1.1.1.21	12	6up3down

In addition, ten unigenes that encoded long-chain acyl-CoA synthetases (LACS, EC: 6.2.1.3), which catalyze the esterification of free fatty acids to CoA upon arrival in the cytoplasm [56], and 15 unigenes that encoded acyl CoA binding protein (ACBP), which binds medium and long-chain acyl-CoA esters with a very high affinity and might function as an intracellular carrier of acyl-CoA esters [57], were also identified. Fig 5 and S5 Table showed that although three unigenes encoding LACS were up-regulated and six were down-regulated, eight unigenes encoding ACBP were up-regulated and three were down-regulated in seeds compared to leaves. This result meant that ACBP might play a critical role in improving oil content in the seeds rather than LACS.

ACCase is a crucial enzyme in *de novo* fatty acid synthesis, and its overexpression could alter the fatty acid composition of seeds and increase the fatty acid content, which would lead to an increased oleic acid content and seeds yield [58, 59]. The transcriptional level of the unigenes encoding ACCase in our transcriptome data was consistent with the reported results. Six unigenes were up-regulated and only two were down-regulated in seeds compared to leaves. The next key enzymes/proteins in fatty acid synthesis are ACP co-factor and the KAS enzymes in FAS. Research on *Brassica juncea* revealed that the functional expression of an ACP from *Azospirillum brasilense* could improve the content of 18:1 and 18:2 in seeds, and enhanced the ratio of monounsaturated (C18:1)/saturated fatty acids and linoleic (C18:2)/linolenic (C18:3) acid. It also reduced erucic acid (C22:1) levels [60]. In our transcriptome, three unigenes that encoded ACP were up-regulated and five were down-regulated. The results suggested that these three unigenes might be very important in the composition and content of fatty acids. We identified three KAS types in plastids (KAS I, KAS II, KAS III). During the first turn of the cycle, the condensation reaction was catalyzed by KAS III which condensed acetyl-CoA with malonyl-ACP to form acetoacetyl-ACP. For the next six turns of the cycle, KAS I catalyzed the condensation reaction to form 16:0-ACP. Finally, KAS II catalyzed 16:0-ACP to elongate to 18:0-ACP. Overexpression of KAS III induced an increase in the levels of 16:0 in tobacco, but reduced the rate of lipid synthesis [61]. Likewise, the suppression of KAS II led to an increase in 16:0 accumulation (53%), but there were deformities in some of the transgenic offspring [62]. Changes to KAS I caused a mutant that had a different polar lipid composition, disrupted embryo development and reduced fatty acid levels (~33.6% of the wild type) in its seeds [63], which suggested that KAS I was also very important to fatty acid synthesis. Unigenes that encoded KAS were almost all up-regulated in seeds compared to leaves in our transcriptome, which indicated that KAS was crucial to the change seen in the quality and content of fatty acids in *B*. *napus* seeds.

### Genes related to TAG and OB biosynthesis

We identified 43 unigenes that encoded seven enzymes involved in the suggested pathway for TAG biosynthesis (Table 6, Fig 5) [3, 64]. Three unigenes that encoded glycerol kinase (GK, EC: 2.7.1.30) and twelve unigenes that encoded glycerol-3-phosphate dehydrogenase (GPDH, EC: 1.1.1.8 1.1.5.3) were identified. They catalyzed the glycerol to glycerol-3-phosphate (G-3-P) step, an initial substrate in the TAG pathway. Then 11 unigenes that encoded the key enzyme of TAG biosynthesis, glycerol-3-phosphate acyltransferase (GPAT, EC: 2.3.1.15; one for GPAT1, two for GPAT2, two for GPAT3, one for GPAT4, two for GPAT5, two for GPAT6 and one for GPAT8), were identified. These enzymes catalyzed the first acylation of G-3-P at the *sn*-1 position to form lysophosphatidic acid (Lyso-PA). The second acylation was catalyzed by 1-acyl-sn-glycerol-3-phosphate acyltransferase (LPAT, EC: 2.3.1.51; four for LPAT1, one for LPAT2, one for LPAT3 and one for LPAT4), to form phosphatidic acid (PA) at the *sn*-2 position of G-3-P. Among these 34 unigenes, 12 unigenes were up-regulated and 12 unigenes were down-regulated in seeds compared to leaves, which showed that they were important in both seeds and leaves. After the second acylation, the dephosphorylation of the resultant PA was catalyzed by phosphatidate phosphatase (PP, EC: 3.1.3.4), a key regulator of lipid homeostasis, to form diacylglycerol (DAG). Two unigenes that encoded PP were all up-regulated, which would increase the TAG substrate levels. The final acylation reaction was that several kinds of enzymes catalyzed DAG to TAG. Three kinds of enzymes, differing in their acyl donor, were identified. One was diacylglycerol O-acyltransferase (DGAT, EC: 2.3.1.20), encoded by five unigenes (two for DGAT1 and three for DGAT2), which catalyzed DAG at the *sn*-3 position using a fatty acyl-CoA molecule. Four unigenes were up-regulated among the five unigenes, which indicated that DGAT was a crucial TAG synthesis enzyme in seeds. The second enzyme was phospholipid diacylglycerol acyltransferase1 (PDAT1, EC 2.3.1.43), encoded by two unigenes, which catalyzed DAG using PC as the acyl donor. But the synthesis of DAG by PDAT1 was dependent on lysophosphatidylcholine acyltransferase (LPCAT, EC: 2.3.1.23) activity to regenerate PC from lyso-PC [65]. It was interesting that one unigene that encoded LPCAT and two unigenes that encoded PDAT1 were down-regulated in seeds compared to leaves in *B*. *napus* according to our transcriptome data, which suggested that the two enzymes might play more roles in TAG synthesis in leaves than seeds. The last enzyme was diacylglycerol cholinephosphotransferase (PDCT, EC: 2.7.8.2), encoded by three unigenes, which catalyzed the transfer of the phosphocholine head-group from PC to DAG, leading to an increase in the desaturation of fatty acids to DAG and subsequently to TAG [66]. Two unigenes were down-regulated, which indicated that PDCT and PDAT1 played important roles in TAG synthesis in *B*. *napus* leaves. Previous studies also demonstrated that ectopic expression of DGAT, a key enzyme regulating the rate of the Kennedy pathway, could improve the oil content in *Arabidopsis*, soybean and maize seeds [67–69]. In addition, phospholipase A2 (PLA2, EC: 3.1.1.4), encoded by two unigenes, was identified and might be involved in membrane lipid synthesis associated with PDAT1 and LPCAT, such as PC to TAG biosynthesis.

**Table 6:** Enzymes related to TAG biosynthesis and metabolism identified by annotation of the *B*. *napus* unigenes.

Symbol	Enzymes/Protein	EC Number	Number of unigenes	Up/Down(numbers of unigenes)
**TAG biosynthesis**				
GK	Glycerol kinase	EC:2.7.1.30	3	1up1down
GPDH	Glycerol-3-phosphate dehydrogenase	EC:1.1.1.8 1.1.5.3	12	3up4down
GPAT1	Glycerol-3-phosphate acyltransferase 1	EC:2.3.1.15	1	1up
GPAT2	Glycerol-3-phosphate acyltransferase 2	EC:2.3.1.15	2	2down
GPAT3	Glycerol-3-phosphate acyltransferase 3	EC:2.3.1.15	2	2up
GPAT4	Glycerol-3-phosphate 2-O-acyltransferase 4	EC:2.3.1.15	1	1down
GPAT5	Glycerol-3-phosphate acyltransferase 5	EC:2.3.1.15	2	1up
GPAT6	Glycerol-3-phosphate acyltransferase 6	EC:2.3.1.15	2	1up1down
GPAT8	Glycerol-3-phosphate acyltransferase 8	EC:2.3.1.15	1	1down
LPAT1	1-acyl-sn-glycerol-3-phosphate acyltransferase 1, chloroplastic	EC:2.3.1.51	4	1down
LPAT2	1-acyl-sn-glycerol-3-phosphate acyltransferase 2	EC:2.3.1.51	1	1up
LPAT3	1-acyl-sn-glycerol-3-phosphate acyltransferase 3	EC:2.3.1.51	1	1up
LPAT4	1-acyl-sn-glycerol-3-phosphate acyltransferase 4	EC:2.3.1.51	1	1down
LPAT5	1-acyl-sn-glycerol-3-phosphate acyltransferase 5	EC:2.3.1.51	1	1up
PP	Phosphatidate phosphatase	EC:3.1.3.4	2	2up
DGAT1	Diacylglycerol O-acyltransferase 1	EC:2.3.1.20	2	1up
DGAT2	Diacylglycerol O-acyltransferase 2	EC:2.3.1.20	3	3up
PDAT1	Phospholipid:diacylglycerol acyltransferase 1	EC:2.3.1.158	2	2down
ACBP	Acyl-CoA-binding protein		15	8up3down
**Acyl editing**				
PLA2	Phospholipase A2	EC:3.1.1.4	2	1up1down
LPCAT	lysophosphatidylcholine acyltransferase	EC:2.3.1.23 2.3.1.67	1	1down
PDCT	Diacylglycerol cholinephosphotransferase	EC 2.7.8.2	3	2down
**TAG metabolism**				
TAGL	Triacylglycerol lipase	EC:3.1.1.3	12	3up4down
MAGL	Monoacylglycerol lipase	EC:3.1-	3	1up1down

Once synthesized, the TAG molecules can be stored in the form of an OB surrounded by a membrane composed of a layer of phospholipids embedded with several proteins, such as oleosin, caleosin and steroleosin, in mature seeds [53, 70]. We identified 16 unigenes that encoded oleosin, three encoding caleosin and two encoding steroleosin (Table 7). Olesosin, which contains a hydrophilic oil body-binding domain flanked by two amphipathic domains, helps stabilize OBs by increasing space bit resistance and charge repulsion, which prevent the fusion of OBs [53, 71]. Caleosin was not only involved in the synthesis and metabolism of OBs, but also plays a role in plant drought tolerance and TAG mobilization during germination, possibly by facilitating interactions with vacuoles [71–73]. Steroleosin, in addition to being an oil body-anchoring domain, might represent a class of dehydrogenases/reductases that may play a role in signal transduction by various sterols [74]. Among the 21 unigenes encoding oil body proteins, only two unigenes were down-regulated (one for olesion and one for caleosin). Among the 19 up-regulated unigenes in seeds, some unigenes were not detected in leaves at the transcriptional level (Table 7). This demonstrated that oleosin, caleosin and steroleosin play crucial roles in the synthesis of OBs in *B*. *napus* seeds, which will help future functional studies of *B*. *napus*.

**Table 7:** Unigenes annotated as oleosin, caleosin and steroleosin and the expression in 25DAP seeds and leaves.

Oil body protein	Unigenes ID	Leaves	Seeds	FDR	Log2FC	Regulated
**Oleosin**	c1584.graph_c0					
	c23541.graph_c0	6.312222768	3.656048324	0.594927299	-0.739596381	down
	c24038.graph_c0	0	372.5670435	2.28E-08	9.015085824	up
	c24532.graph_c0	0	725.9146663	2.86E-11	11.44138334	up
	c28274.graph_c0	0	7990.140177	1.11E-16	15.82084718	up
	c29002.graph_c0	0	2.385682868	0.033975201	3.61024562	up
	c29397.graph_c0	0	14.47071134	1.74E-05	6.580151515	up
	c32762.graph_c0	0.533087123	109.6065151	6.50E-07	7.311413204	up
	c32777.graph_c0	0.536044935	4314.157298	2.40E-13	12.67767571	up
	c33249.graph_c0	2.651180655	7784.451408	7.38E-12	11.42973502	up
	c33254.graph_c0	0	1281.10938	3.30E-13	13.06117547	up
	c33463.graph_c0	0	42.25251576	3.73E-07	7.99515731	up
	c35049.graph_c0	0	124.6277195	3.14E-09	9.736084183	up
	c37562.graph_c0	1.046391746	18864.00137	6.66E-15	13.95741831	up
	c49494.graph_c0					
	c52336.graph_c0					
**caleosin**	c33604.graph_c0	101.6787801	58.06664131	0.833270798	-0.793341799	down
	c34268.graph_c0	0	1935.17207	1.15E-14	14.27874692	up
	c46025.graph_c0	0	103.2154874	1.64E-07	8.296078219	up
**steroleosin**	c37523.graph_c0	0	270.6438792	4.88E-11	11.24821215	up
	c40308.graph_c0	0	464.4814328	2.25E-12	12.36463432	up

### Genes related to fatty acid desaturation

Fatty acid desaturation contains two steps. the first step is the formation of monounsaturated fatty acids from saturated fatty acids in plastids, which is catalyzed by AAD [75]. The second step is the formation of unsaturated bonds on the monounsaturated fatty acids at specifically defined positions (△12, △15 or △16), which is catalyzed by enzymes located on the membranes of the endoplasmic reticulum and chloroplast [76], including △12(w6)-desaturase (FAD2 and FAD6, EC: 1.14.19.-), which desaturates oleic acid (18:1) to form linoleic acid (18:2), and △15(w3)-desaturase (FAD3, FAD7 and FAD8, EC: 1.14.19.-), which further desaturates linoleic acid (18:2) to form α-linolenic acid (18:3). Besides these desaturations, we also identified palmitoyl-monogalactosyldiacylglycerol delta(δ)-7 desaturase (FAD5, EC: 1.14.19.-), which affects the accumulation of 16:3Δ^7,10,13^ by catalyzing 16:0 MGDG to form 16:1 MGDG at position (△7) in leaves [3, 77, 78]. We found that three unigenes encoded AAD, one encoded FAD2, one encoded FAD3, four encoded FAD5, two encoded FAD6, six encoded FAD7 and two encoded FAD8 (Table 5). The unigenes that encoded AAD, FAD2 and FAD3 were up-regulated, which showed that these unigenes might play an important role in oleic acid (18:1) biosynthesis in seeds, for the unigenes encoded FAD2 and FAD3 were identified in endoplasmic reticulum. Six unigenes out of the seven that encoded FAD6, 7 and 8, and one unigene that encoded FAD5 were down-regulated, which could explain the high PG, MGDG, DGDG and SQDG contents in leaves for these unigenes were identified in chloroplastic, which are produced by the synthesis pathways for prokaryotic galactolipid, sulfolipid and phospholipid [3]. These results will help us to further explore lipid synthesis by leaves.

### Genes related to the catabolism pathways for TAGs and fatty acids

The long-chain, insoluble TAGs are hydrolyzed in two steps. First, the TAGs are catalyzed by triacylglycerol lipase (TAGL, EC: 3.1.1.3) to hydrolyze the ester bonds that link fatty acyl chains to the glycerol backbone by releasing free fatty acids from DAG and TAG. The last ester bond is hydrolyzed by monoacylglycerol lipase (MAGL, EC: 3.1.-) [3]. Twelve unigenes that encoded TAGL and three encoding MAGL were identified in the *B*. *napus* transcriptome. We found that there were four down-regulated and three up-regulated unigenes for TAGL, and one down-regulated and one up-regulated unigene for MAGL (Table 6), which showed that these unigenes in both leaves and seeds were crucial for lipid degradation. The second step in TAG catabolism is the catabolism of fatty acids to form acetyl-CoA, which is further broken down by oxidation or other metabolic pathways [79]. According to the KEGG pathway assignment and annotation of the unigenes in the transcriptome, 82 unigenes that encoded eight kinds of enzymes related to fatty acid catabolism were identified; three key enzymes were acyl-CoA oxidase (ACOX, EC: 1.3.3.6), enoyl-CoA hydratase/3-hydroxyacyl-CoA dehydrogenase (MFP2, EC: 4.2.1.17 1.1.1.35 1.1.1.211) and acetyl-CoA acyltransferase (ACAT, EC: 2.3.1.16), which were encoded by 11, 12 and one unigenes, respectively (Table 5). The acetyl-CoA generated by fatty acid catabolism is used to produce energy for the cell via the citrate cycle or participates in TAG biosynthesis.

TAG and fatty acid catabolism proceeds in an opposite direction to their synthesis. So, the way to increase the accumulation of lipids may be to suppress the catabolism of TAG and fatty acids, which would improve the quality of *B*. *napus*.

### Detection of TFs involved in lipid synthesis

Many TFs were involved in the synthesis and deposition of seed oil, such as LEC1, LEC2, ABI3, WRI1 and FUS3 (http://lipidlibrary.aocs.org/plantbio/transfactors/index.htm) [27, 28]. In this study, we identified that 3387 unigenes annotated with 1122 independent *Arabidopsis* TFs coding sequences belonged to 49 known TF families [47]. We found that the largest number of unigenes (667) was annotated to the Trihelix family, followed by the C2H2 family (456) (S6 Table). We identified 27 unigenes that encoded 11 TFs that are involved in oil biosynthesis according to research by Fobert (Table 8). These 11 TFs were ABI3, LEC1, WRI1, ADOF1, EMF2, AP2, LEC2, FUS3, GL2, HSI2-L1 and HSI2, and they might play a more important role in the synthesis of seed oil than in leaf oil. However, there were no unigenes that showed homology to L1L, PKL, FIE or SWN, which indicated that these TFs did not have much of a role in oil synthesis. To further understand the function of these 11 TFs, we analyzed the expression of the unigenes encoding these TFs (Table 8). We found that nearly all the unigenes were up-regulated in seeds compared to leaves, except for one ADOF1 unigene and one AP2 unigene. Among the up-regulated unigenes, the unigenes encoding ABI3, LEC1, FUS3 and GL2 in leaves had no expression at the transcription level, which revealed that these TFs played an important role in seed oil synthesis, but were probably not involved in oil synthesis in leaves. WRI1 was much more highly expressed in seeds than in leaves, which suggested that it had an extremely important role in oil synthesis, which was consistent with the function of WRI1 during fatty acid biosynthesis and photosynthesis, where it regulates the expression of GT1-element and/or GCC-box containing genes [80]. We also performed a wide expression analysis of all the transcription factor families (Table 9). Among these transcription factor families, ABI3VP1, AtRKD, CPP, E2F-DP, GRF, JUMONJI, MYB-related, PHD and REM may play an important role in lipid biosynthesis by seeds because the up-regulated unigenes in these transcription factor families made up a larger percentage (over 90% in all expressed unigenes) than the down-regulated unigenes (Table 9). This result showed that the unigenes in these TF families might be involved in or even contribute to the oil synthesis in seeds, which would lay a foundation for further research on transcription factor regulation during lipid biosynthesis. In summary, these analyses could provide further information about the regulation mechanism underlying TFs’ roles in oil synthesis.

**Table 8:** The relative expression of unigenes identified as the TF involved in oil synthesis.

TF name	Unigenes ID	Leaves	Seeds	FDR	Log2FC	Regulated
ABI3(Abscisic Acid Insensitive 3)	c42986.graph_c0	0	53.901553	3.05E-10	10.58245	up
LEC1(Leafy cotyledon1)	c33792.graph_c0	0	22.168763	2.17E-06	7.3483149	up
WRI1(Wrinkled1)	c40435.graph_c0	1.1940946	73.933088	3.01E-05	5.8758729	up
ADOF1(Arabidopsis Dof Zinc Finger Protein 1)	c34995.graph_c0	75.485511	24.245782	0.5465201	-1.62022	down
	c22022.graph_c0					
EMF2(Embryonic Flower 2)	c23937.graph_c0	0.2626646	3.7354577	0.0396252	3.1316306	up
	c43162.graph_c0	6.0134657	19.844284	0.4507925	1.725307	up
AP2(APETALA2)	c42473.graph_c0	11.026932	13.492089	0.7413012	0.3010018	up
	c2509.graph_c0	3.7956765	0.3490075	0.0485946	-2.682913	down
LEC2(Leafy cotyledon2)	c57560.graph_c0					
	c55216.graph_c0					
FUS3(Fusca3)	c33720.graph_c2	0	10.742026	0.0004825	5.3306493	up
	c38470.graph_c0	0	30.22454	4.49E-08	8.767464	up
GL2(GLABRA2)	c26728.graph_c0	0	7.737113	0.0016967	4.8439302	up
	c40770.graph_c0	0	6.9014965	0.0001735	5.7197442	up
	c40770.graph_c1	0	7.5332505	9.66E-05	5.9406611	up
HSI2-L1/HSL1(HSI2-Like 1)	c32344.graph_c0					
	c45245.graph_c0	0.9631632	6.6234755	0.0779525	2.7608182	up
	c45808.graph_c0	1.2847384	9.3659689	0.0664691	2.8455825	up
	c43966.graph_c0	7.6220108	8.9851268	0.7515839	0.2482162	up
HSI2(High-Level Expression of Sugar-Inducible Gene 2)	c27173.graph_c0					
	c28819.graph_c0	0.1216735	4.2177701	0.0239492	3.5552057	up
	c32124.graph_c0	0.3471751	10.723232	0.0031922	4.2128966	up
	c45955.graph_c0	2.114279	12.57168	0.1269483	2.5626317	up
	c28424.graph_c0	0.9865357	4.1651317	0.3120835	1.7648368	up
	c27807.graph_c0	0	5.1606096	0.0083859	4.2048549	up
	c41698.graph_c0	0.2267668	3.8850453	0.0053936	3.8479633	up

**Table 9:** The expression distribution of unigenes in transcript factor families in *B*.*napus*.

TF families	Numbers of expressed unigenes	Numbers of up-reuglated unigenes against leaves	Numbers of down-reuglated unigenes against leaves	Percentage of up-regulated unigenes against leaves	Percentage of down-regulated unigenes against leaves
ABI3VP1	18	17		94.44%	5.56%
Alfin-like	7	5	2	71.43%	28.57%
AP2-EREBP	88	38	50	43.18%	56.82%
ARF	31	23	8	74.19%	25.81%
ARID	4	3	1	75.00%	25.00%
ARR-B	22	13	9	59.09%	40.91%
AtRKD	1	1	0	100.00%	0.00%
BBR/BPC	7	3	4	42.86%	57.14%
bHLH	178	75	103	42.13%	57.87%
bZIP	60	34	26	56.67%	43.33%
BZR	9	4	5	44.44%	55.56%
C2C2-CO-like	30	6	24	20.00%	80.00%
C2C2-Dof	25	14	11	56.00%	44.00%
C2C2-Gata	22	10	12	45.45%	54.55%
C2C2-YABBY	4	3	1	75.00%	25.00%
C2H2	379	222	157	58.58%	41.42%
C3H	261	141	120	54.02%	45.98%
CAMTA	27	17	10	62.96%	37.04%
CCAAT-HAP2	6	4	2	66.67%	33.33%
CCAAT-HAP3	5	2	3	40.00%	60.00%
CCAAT-HAP5	8	7	1	87.50%	12.50%
CPP	5	5	0	100.00%	0.00%
E2F-DP	7	7	0	100.00%	0.00%
EIL	6	3	3	50.00%	50.00%
G2-like	35	21	14	60.00%	40.00%
GeBP	12	7	5	58.33%	41.67%
GRAS	33	12	21	36.36%	63.64%
GRF	10	10	0	100.00%	0.00%
Homeobox	87	58	29	66.67%	33.33%
HRT	1	0	1	0.00%	100.00%
HSF	28	15	13	53.57%	46.43%
JUMONJI	4	4	0	100.00%	0.00%
MADS	34	19	15	55.88%	44.12%
MYB	49	28	21	57.14%	42.86%
MYB-related	2	2	0	100.00%	0.00%
NAC	75	46	29	61.33%	38.67%
NLP	8	5	3	62.50%	37.50%
Orphan	3	0	3	0.00%	100.00%
PHD	19	19	0	100.00%	0.00%
RAV	3	0	3	0.00%	100.00%
REM	27	26	1	96.30%	3.70%
SBP	9	6	3	66.67%	33.33%
TCP	49	21	28	42.86%	57.14%
Trihelix	518	266	252	51.35%	48.65%
TUB	9	6	3	66.67%	33.33%
VOZ	4	3	1	75.00%	25.00%
Whirly	2	0	2	0.00%	100.00%
WRKY	182	113	69	62.09%	37.91%
ZF-HD	18	12	6	66.67%	33.33%
CCAAT-DR1	0	0	0	0	0

### Real-time PCR analysis of selected TFs

**Fig 6:**
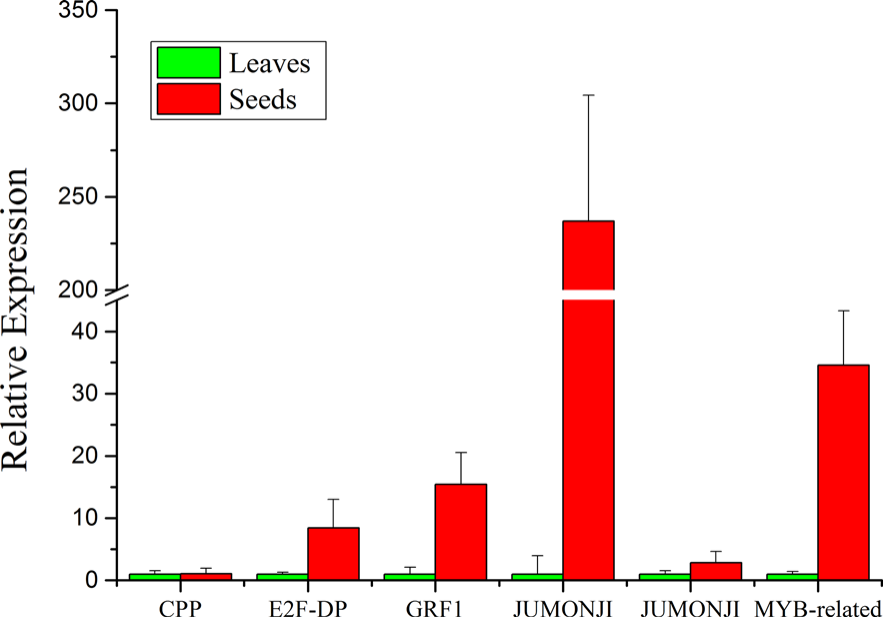
Relative expression of selected unigenes for transcript factors between leaves and developing seeds.

To confirm the expression difference of identified transcript factors in seeds and leaves, nine unigenes were selected for qRT-PCR analysis (Fig 6). Among these nine unigenes, three unigenes encoding transcript factors LEC1, HSI2 and REM16 were detected no expression in leaves, and only showed expression in seeds, indicating that these three transcript factors played more important roles in seeds. The other unigenes for transcript factors CPP, E2F-DP, GRF1, JUMONJI and MYB-related were up-regulated in seeds, and the most increased was the unigene JUMONJI, followed by MYB-related, GRF, E2F-DP, JUMONJI and CPP. Because the main metabolisms in seeds are fatty acids and lipids synthesis metabolism, so the high expression level of these unigenes for transcript factors might be involved in the fatty acids and lipids synthesis in seeds. The results of qRT-PCR analysis confirmed the transcriptome data. These TFs might provide the new clue for understanding the mechanism of fatty acids and lipids biosynthesis in seeds.

## Conclusion

Although the sequencing of the whole *B*. *napus* genome has been finished [38], which could provide huge genomic information for scientific research, the regulation of lipids synthesis between leaves and seeds in *B*. *napus* was still unclear. This study has revealed how the genes involved in the biosynthesis and metabolism of lipids were regulated by analyzing the *B*. *napus* seeds and leaves transcriptome. We found 47216 unigenes. Information about these unigenes will aid future genomic gene expression assay research and can serve as a reference transcriptome for future *B*. *napus* experiments. We identified the unigenes that encoded key enzymes and TFs that were involved in the metabolic pathways for fatty acids, and TAG biosynthesis and metabolism. We also found some genes, TFs and proteins that played extremely important roles in the accumulation of fatty acids and lipids when we compared the seeds with the leaves, such as ACCase, HAD, KASII, PP, DGAT1,2, ABI3, LEC1, WRI1, FUS3, oleosin, caleosin and steroleosin. These results will offer molecular guidance to further, more targeted experiments on *B*. *napus*. We identified some new TFs that might promote the lipid biosynthesis metabolic pathway in seeds by the transcriptome and qRT-PCR analysis, such as JUMONJI, MYB-related, GRF, E2F-DP, CPP and REM16. The gene expression regulation analysis revealed the cause of the different lipid contents between seeds and leaves and how they have evolved different functions. This study has provided insights into the molecular mechanism underlying lipid biosynthesis, and has laid the foundations for further improvements to seeds lipids through genomics research.

## Supporting Information

S1 Table Pathway annotation of unigenes from *B*. *napus*. (XLSX)

S2 Table The GO classification of DEG unigenes. (XLSX)

S3 Table The COG classification of DEG unigenes. (XLSX)

S4 Table Detailed information about the enzymes/proteins related to fatty acid biosynthesis and metabolism identified by annotation of the *B*. *napus* unigenes. (XLSX)

S5 Table Detailed information about the enzymes related to TAG biosynthesis and metabolism identified by annotation of the *B. napus* unigenes. (XLSX)

S6 Table Putative transcription factors encoding unigenes in *B*. *napus*. (XLSX)

S7 Table Gene-specific primers used for gene expression analysis by quantitative real-time PCR. (XLSX)
