# Correction: Transcriptome Analysis Comparison of Lipid Biosynthesis in the Leaves and Developing Seeds of *Brassica napus*

**DOI:** 10.1371/journal.pone.0130067

**Published:** 2015-06-09

**Authors:** Jie Chen, Ren-Ke Tan, Xiao-Juan Guo, Zheng-Li Fu, Zheng Wang, Zhi-Yan Zhang, Xiao-Li Tan

The Data Availability statement for this paper is incorrect. The correct statement is: The unigenes information was deposited in the Sequence Read Archive (SRA) database in NCBI (Accession number, SRP055854).

In the subsection Analysis of transcriptome sequencing results of the Materials and Methods section, the final sentence of the first paragraph references the incorrect accession number. The complete, correct sentence is: The unigenes information was deposited in the Sequence Read Archive (SRA) database in NCBI (Accession number, SRP055854).
